# Succession and Colonization Dynamics of Endolithic Phototrophs within Intertidal Carbonates

**DOI:** 10.3390/microorganisms8020214

**Published:** 2020-02-05

**Authors:** Daniel Roush, Ferran Garcia-Pichel

**Affiliations:** 1 School of Life Sciences, Arizona State University, Tempe, AZ 85282, USA; dwroush@asu.edu; 2 Center for Fundamental and Applied Microbiomics, Biodesign Institute, Arizona State University, Tempe, AZ 85282, USA

**Keywords:** bioerosion, anoxygenic phototroph, microbiome, euendolith

## Abstract

Photosynthetic endolithic communities are common in shallow marine carbonates, contributing significantly to their bioerosion. Cyanobacteria are well known from these settings, where a few are euendoliths, actively boring into the virgin substrate. Recently, anoxygenic phototrophs were reported as significant inhabitants of endolithic communities, but it is unknown if they are euendoliths or simply colonize available pore spaces secondarily. To answer this and to establish the dynamics of colonization, nonporous travertine tiles were anchored onto intertidal beach rock in Isla de Mona, Puerto Rico, and developing endolithic communities were examined with time, both molecularly and with photopigment biomarkers. By 9 months, while cyanobacterial biomass and diversity reached levels indistinguishable from those of nearby climax communities, anoxygenic phototrophs remained marginal, suggesting that they are secondary colonizers. Early in the colonization, a novel group of cyanobacteria (unknown boring cluster, UBC) without cultivated representatives, emerged as the most common euendolith, but by 6 months, canonical euendoliths such as *Plectonema* (*Leptolyngbya*) sp., *Mastigocoleus* sp., and Pleurocapsalean clades displaced UBC in dominance. Later, the proportion of euendolithic cyanobacterial biomass decreased, as nonboring endoliths outcompeted pioneers within the already excavated substrate. Our findings demonstrate that endolithic cyanobacterial succession within hard carbonates is complex but can attain maturity within a year’s time.

## 1. Introduction

The endolithic microbiome of intertidal carbonate rocks has been the subject of intensive study since the 1800s [1,2], with a main focus on the characterization of bioerosive agents within these communities. The agents, boring organisms referred to as euendoliths, excavate the rock substrate and create pore spaces for their own growth. Le Campion-Alsumard and colleagues [3,4] first examined succession and colonization by microscopic inspection in order to better understand the ecological principles that drive euendolith community formation. As concern for coral destruction rose in the 1990s, others [5,6,7,8,9,10,11] applied the same procedures to understand these dynamics and mitigate bioerosion in reef ecosystems. These studies on porous, biogenic carbonates from coral skeletons showed swift initial colonization by euendolithic algae, with successional changes occurring within months and communities reaching maturity after a year. Kiene [10], Gektidis [9] and Chacón et al. [12] examined hard mineral carbonates as well, finding that euendolithic cyanobacteria, not algae, were the dominant boring organisms there and that hard substrates led to more diverse cyanobacterial populations than those of corals. 

Although early research was informative in identifying and characterizing major euendolithic players, the use of morphological characterization alone has been found to underestimate microbial diversity in endolithic cyanobacterial communities [12,13]. Indeed, in the case of marine carbonate communities, high-throughput amplicon sequencing has demonstrated that morphology-based studies can underrepresent cyanobacterial diversity estimates by factors of 10 to 100 [12,14,15]. Early research identified three major morphotypical groups of euendolithic cyanobacteria. One is represented by the thin, filamentous, *Leptolyngbya*-like organisms most commonly assigned to *Plectonema terebrans* (or *Leptolyngbya terebrans*), which are typically one of the most abundant euendolith morphotypes, at times exceeding 80% of total euendolithic biovolume [6]. Unfortunately, no 16S rRNA gene sequence of *P. terebrans* has been obtained from cultures, making it impossible to identify it with certainty in molecular surveys. Environmental sequences best matching *Halomicronema* and *Leptolyngbya* species have been tentatively suggested to represent the elusive *P. terebrans* [14]. The second group corresponds to the species *Mastigocoleus testarum,* which is characterized by a complex, true-branching filamentous morphology, making it easily identifiable from microscopic examination. It has been recently redescribed on the basis of a polyphasic approach based on strain BC008, showing congruency between molecular and traditional approaches [16], has served as a model to elucidate the physiological mechanism of boring [16,17,18], and is the first euendolith whose genome has been fully sequenced [19]. *Mastigocoleus testarum* is one of the earliest colonizers in soft carbonates, being found as early as one week after initial exposure [3,11,20]. A third, diverse group includes several members of the order Pleurocapsales in the genera *Hyella, Solentia*, *Hormathonema*, and the recently described *Candidatus* Pleuronema. Members of the Pleurocapsales typically act as pioneer borers but can bore only to shallow depths and are easily preyed upon by grazers, leading to low abundance in mature communities [6,7,11,20]. 

Through comprehensive, high-throughput molecular surveys, we recently found a diverse phototrophic community in the endolithic habitat of coastal hard carbonates, which included four distinct anoxygenic phototrophic bacterial (APB) groups. The most dominant APBs were members of the Chloroflexales (green nonsulfur bacteria) [21,22,23] and *Erythrobacter* (aerobic anoxygenic phototrophs) [24,25]. APBs could comprise upwards of 80% of the total phototroph community [15] in some samples. Our findings broadened the known habitats for APBs and suggested that some microscopic characterizations of endolithic thin filamentous organisms (*Plectonema*-like) may have in fact been APBs. Thus, APBs could be euendolithic in nature, potentially upending the long established understanding of endolith ecology by broadening the pool of possible pioneer organisms and boring mechanisms. 

Therefore, to provide new molecular insights into euendolith colonization and succession and to attempt to answer questions that arose from our prior work, we set up a colonization experiment in the intertidal zone of Playa Ulvero, Isla de Mona, Puerto Rico. We anchored nonporous travertine (a dense, compact form of calcium carbonate) tiles onto beach rock 5 m from shore and collected samples every 3 months over a 9-month period. Our study had four specific aims: (1) to elucidate APB colonization timing to identify if APBs are pioneer organisms with the ability to bore; (2) to examine cyanobacterial euendolith colonization and succession using molecular methods; (3) to measure the colonization dynamics of the *Leptolyngbya*-like (*Plectonema*), *Mastigocoleus*-like, and Pleurocapsalean euendolithic cyanobacterial groups; and (4) to compare colonization progress to previously described steady-state climax communities of similar geological composition and geographic location in order to gauge community maturity 

## 2. Materials and Methods

### 2.1. Tile Placement and Sample Collection

**Figure 1:**
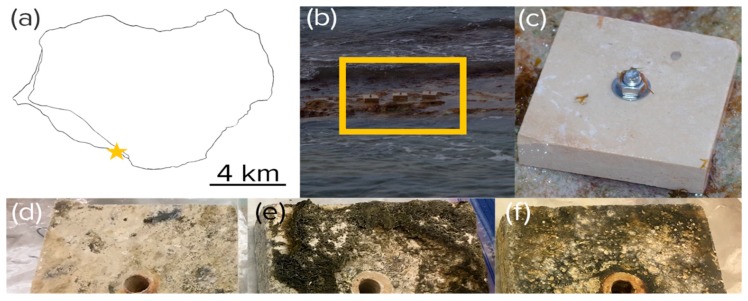
Experimental tile placement. (**a**) Location near Playa Uvero (yellow star) on Isla de Mona, Puerto Rico. (**b**) Anchoring on a stretch of intertidal beach rock (yellow box) as seen at low tide. (**c**–**f**) Aspect of virgin (**c**) and exposed tiles harvested after harvested after 3 (**d**), 6 (**e**), and 9 months (**f**). Part of the growth observable in the pictures was epilithic in nature.

Commercial, 4 inches wide, 1.5 inches thick travertine square tiles, were anchored onto intertidal beach rock some 5 m from the high-tide shoreline at Playa Uvero on Isla de Mona, Puerto Rico, (18°03′36.2″ N 67°54′21.8″ W) (Figure 1) after having received permits from the Departamento de Recursos Naturales y Ambientales (Commonwealth of Puerto Rico). Tiles were fastened to the beach rock using a combination of Red Head 5” × 3/8” 316 Stainless Steel Wedge Anchors and JB Weld Waterweld putty. Three tiles were sacrificially collected every three months, air-dried and shipped, reaching the laboratory in less than a week, and then stored on arrival at −80 °C until analysis.

### 2.2. Endolithic Community DNA Extraction

**Figure 2:**
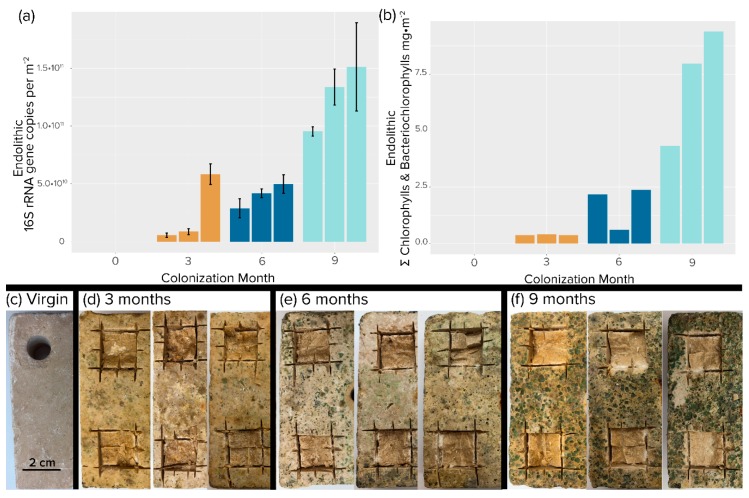
Endolithic colonization of travertine tiles. (**a**) Areal concentration of 16S rRNA gene copies. Each bar is an independent replicate. Error bars are from biological replicates. (**b**) Areal concentration of total photosynthetic chlorins (chlorophylls plus bacteriochlorophylls). Single determinations were carried out for each replicate tile. (**c**–**f**) Photographic evidence of colonization after removal of epilithic biomass by brushing. (**c**) Initial, virgin tile. Excising squares were samples used for analyses.

Tiles were vigorously brushed with sterile toothbrushes and sterilized seawater to remove epilithic biomass. To ensure a consistent sampling effort, each tile was sampled four times in 2 cm by 2 cm squares, 1 cm from the edge of the tile (sampling is shown in Figure 2d–f). Sampled fragments were ground in sterile mortars following the protocol described in Wade and Garcia-Pichel 2003 [26], and 0.5 g of powered rock was used as the input material for a MoBio PowerPlant Pro DNA extraction kit (Mo Bio Laboratories, Inc., Carlsbad, CA, USA) following the protocol provided, except that, before the first lysis step, the contents of the bead tubes were homogenized horizontally at 2200 rpm for 10 min, and, additionally, subjected to seven freeze–thaw cycles using liquid nitrogen to ensure full disruption of bacterial membranes.

### 2.3. Quantitative PCR of 16S rRNA Gene Content

In order to quantify the number of 16S rRNA gene copies in the extracts, quantitative real-time PCR was conducted using universal V3 16S rRNA gene primers 338F (5’- ACTCCTACGGGAGGCAGCAG-3’) and 518R (5’-GTATTACCG CGGCTGCTGG-3’). PCRs were performed in triplicate using Sso Fast mix (Bio-Rad, Hercules, CA, USA) following Couradeau et al. [27]. Following quantification, triplicate 16S rRNA gene counts were averaged and then converted to counts per square meter using the surface area of the tile analyzed. The total counts per square meter were then multiplied by the associated proportional abundance of any clade of interest in order to obtain absolute population size for that clade. Separate biological replicates (i.e., tiles) were then averaged.

### 2.4. 16S rRNA Gene Library Preparation and Illumina Sequencing

Amplicon sequencing of the V3–V4 variable region of the 16S rRNA gene was performed using the universal bacterial PCR primers 341F (5’-CCTACGGGNGGCWGCAG) [28] and 806R (5’-GGACTACVSGGGTATCTAAT) [29]. PCR amplifications were done in triplicate, then pooled and quantified using Quant-iT™ PicoGreen® dsDNA Assay Kit (Invitrogen). Two hundred forty nanograms of DNA per sample were pooled and then cleaned using QIA quick PCR purification kit (QIAGEN). The PCR pool was quantified by Illumina library Quantification Kit ABI Prism® (Kapa Biosystems). DNA pool was determined and diluted to a final concentration of 4 nM then denatured diluted to a final concentration of 4 pM with a 30% of PhiX. Finally, the DNA library was loaded in the MiSeq Illumina sequencer using the chemistry version 3 (2 × 300 paired-end) and following the guidelines of the manufacturer.

### 2.5. Data Analysis Pipeline 

Raw sequences were processed using the QIIME2 2018.2 analysis pipeline [30]. Demultiplexed sequences were imported into QIIME2 and processed using the DADA2 [31] denoised-paired plugin with the following parameters: trunc_len_f:280, trunc_len_r:235, trim_left_f:20, trim_left_r:25, and max_ee:8, so as to obtain amplicon sequence variants (ASVs). After resolving ASVs, any sequences found in the control tile extracts (uncolonized tiles) were filtered from the final feature table. Sequencing depth of the experimental tiles ranged from 21,897 to 180,168 (post filtering), and alpha-rarefaction analysis indicated that all samples had reached convergence (Figure S1). In order to conduct diversity analysis, representative sequences were aligned using MAFFT7 [32], and a phylogenetic tree was generated using FastTree [33]. Diversity metrics were calculated using the core-metrics-phylogenetic plugin, including Weighted and Unweighted UniFrac metrics [34]. ASVs were initially classified using the classify-sklearn plugin, (Available online: https://github.com/qiime2/q2-feature-classifier) with a Green Genes 13_8 [35] based classifier. The feature table was then exported, and differential abundance analysis was conducted using the QIIME1 [36] plugin *differential_abundance.py* and the DESeq2 algorithm [37]. PCoAs were generated using the vegan package [38], and graphics were created using R [39] and the ggplot2 package [40]. Statistical analyses were conducted either using R (Student’s *t*-test) or within Qiime2 (Kruskal–Wallis, PERMANOVA). 

### 2.6. Cyanobacterial ASV Classification

To identify key euendolithic cyanobacterial clades, the representative sequence output from QIIME2 was filtered to only include cyanobacterial sequences (plastids were removed). These comprised at least 95% of the total number of reads within each sample. Next, the sequences were aligned to the Cydrasil reference alignment [41] using PaPaRa [42], and placed into the Cydrasil reference tree using the Evolutionary Placement Algorithm (based on the maximum-likelihood model) feature of RAxML8 [43]. The output was visualized using the ITOL3 website [44]. An ASV was considered a likely euendolith if it was placed on a branch containing only known euendolithic cyanobacteria with a >70% certainty. Biomass was calculated for each tile by multiplying the total relative abundance of the cluster by the total areal concentration of 16S rRNA genes in that sample. Then, biological replicates for each time point were averaged and graphed using R and ggplot2. 

### 2.7. Steady-State Climax Community Comparisons

Three natural substrate samples from Couradeau et al. [14] and Roush et al. [15] (samples denoted as H001-H003 in SRA) were used as proxies for steady-state climax communities for comparison of colonization progress. The samples were chosen based upon their geographic proximity to the tile placement location and their similar geological composition (calcite). The raw sequencing data was processed using the same parameters and pipeline as described above. Pigment analysis was conducted in the same manner as for the tiles. 

### 2.8. Unknown Boring Cluster (UBC) Phylogenetic Tree 

In order to assess the nearest neighbors of the unknown boring cluster, a multiple sequence alignment (MSA) was generated using SSU-Align [45]. The MSA was comprised of the three most differentially abundant ASVs identified from DESeq2 and EPA placement analysis, the nearest sequences from Cydrasil, and the top seven most similar NCBI nr database sequences identified using BLAST [46]. The resulting alignment of 398 sequences was then used as input into RAxML8 [47] to generate a phylogenetic tree using the rapid-bootstrap algorithm with 1000 bootstraps and the GTR GAMMA model. The remaining ASVs were then checked using BLAST and the nr database for proximity to the resulting clade.

### 2.9. Pigment Extraction and Analysis 

In order to extract lipid-soluble pigments, the remaining powdered sample (the same samples used for DNA extraction) was suspended in 7:2 acetone:methanol solvent and sonicated twice for 30 s in an ice bath in the dark. Extracts were centrifuged at 2100× *g* for 10 min, decanted, and the supernatant filtered through a 0.22 µm nylon filter. These steps were repeated and the supernatants were pooled until the extract was devoid of color. The resulting extract was then evaporated under a N_2_ stream in the dark and resuspended in 200 µL of HPLC-grade acetone. HPLC analysis was conducted on a Waters Alliance e2695 HPLC with an inline Waters 2998 photodiode array detector, using a protocol adapted from Frigaard et al. [48] for use on a CORTECS C18 4.6 mm × 150 mm (90 Å pore size, 2.7 µm particles) column. Separation was performed as follows: the initial solvent gradient composed of ethyl-acetate:methanol:acetonitrile:water in a 21:23.9:47.6:7.5 ratio by volume and linearly changed to 30:20:50:0 ratio by volume in 13.43 min, held for 3.87 min, and then immediately returned to the initial ratio (21:23.9:47.6:7.5 by volume) and held for 5.7 min. Total runtime per sample was 23 min, at a flow rate of 2 mL min^–1^ and column temperature of 30 °C. Pigment identification was done by comparison of retention time and spectrum against standards of Chl *a* and BChl *a* obtained from Sigma Aldrich. All other pigments were identified from known spectra [49]. Injected pigment mass was calculated from the chromatogram using the equation m = FA (*e*_m_ d)^−1^, where m is the mass of BChl or Chl in milligrams, *F* is the solvent flow rate (1 mL min^−1^), A is the peak area (in Au), *e*_m_ is the extinction coefficient in L mg^−1^ cm^−1^, and d is the path length of the PDA detector (1 cm). Extinction coefficients were taken from Ley et al. 2006 [50]. 

### 2.10. Data Availability 

Isla de Mona steady-state climax community raw sequencing data is deposited under NCBI BioProject PRJNA603780. Raw sequencing data from the experimental tiles is deposited under NCBI BioProject PRJNA596277.

## 3. Results

### 3.1. Endolithic Bacterial and Phototrophic Growth

**Figure 3:**
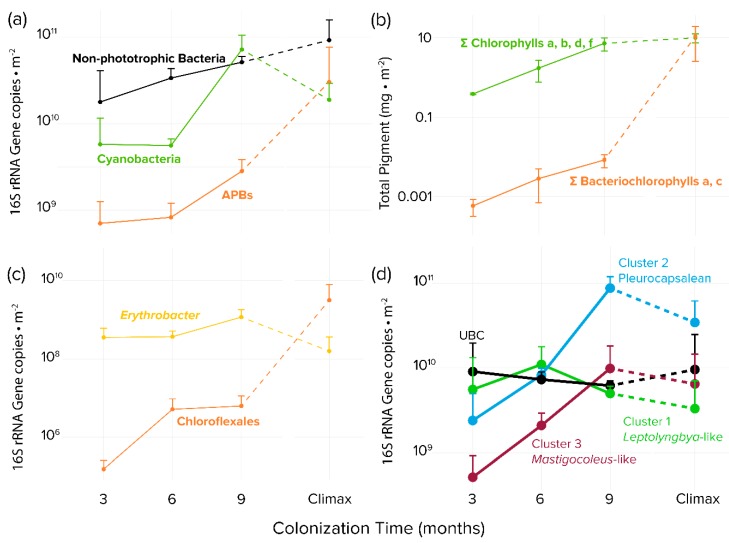
Time series of bacterial biomass proxies detected in colonized tiles and steady-state climax communities by guild or taxon. (**a**) Areal concentrations of 16S rRNA gene copies based on quantitative PCR and high-throughput sequencing phylogenetic assignments (**b**) Areal photosynthetic chlorins as biomarkers for oxygenic phototrophs (total chlorophylls) or APB (total bacteriochlorophylls) (**c**) areal population size of APB clades *Erythrobacter* spp. and Chloroflexales based on quantitative PCR and high-throughput sequencing phylogenetic assignments. (**d**) Endolithic colonization dynamics of specific microboring cyanobacterial clades, based on qPCR and bioinformatic placement of high-throughput environmental sequences using the Cydrasil cyanobacterial reference tree and database. Error bars are for biological sample triplicates.

Visual inspection of the colonized tiles showed a marked increase in both pigmentation and erosion with time (Figure 2c–f). The tiles sustained both nonphototrophic and phototrophic bacterial growth over the 9-month exposure period. Bacterial biomass increased at an average rate of 3 × 10^10^ 16S rRNA gene copies per m^−2^ month^−1^, reaching a mean value of 1.1 × 10^11^ 16S rRNA gene copies per m^−2^ at 9 months (Figure 3a). As expected, phototroph colonization followed a similar trend with photopigment content increasing at a rate of 2.3 mg m^−2^ month^−1^, reaching an average value of 7.22 mg m^−2^ by 9 months. (Figure 3b), at which point 16S rRNA gene counts were not significantly different from those found in steady-state climax communities described by Couradeau et al. [14] and Roush et al. [15] (Student’s *t*-test, *p* < 0.05). While total chlorophyll pigment concentrations were not significantly different between 9 months and steady-state climax communities either, cyanobacteria-specific counts were actually higher at 9 months than in steady-state climax communities.

### 3.2. Incidence of Anoxygenic Phototrophs

APB abundance measured by bacteriochlorophylls increased with time but trailed in concentration by some two orders of magnitude to cyanobacterial abundance measured by chlorophylls during the colonization period. This situation obviously changed significantly later during succession, as bacteriochlorophylls were statistically as abundant as chlorophylls when compared to steady-state climax communities (Figure 3b). The magnitude of the difference between APB and cyanobacteria was less marked, but still very significant, when measured by 16S rRNA gene abundance (Figure 3a). By using this metric it was obvious that although APB trailed cyanobacteria during the colonization period, they eventually matched and even exceeded cyanobacteria in steady-state climax communities. We found very differing dynamics between populations of relevant APB groups: while Chloroflexales were only present in very small quantities during early phases (Figure 3c) and reached only 6.2 × 10^6^ 16s rRNA gene copies per m^−2^ after 9 months, *Erythrobacter* abundance was stable throughout the colonization, with an average of 1.2 × 10^9^ 16S rRNA gene copies per m^-2^ at 9 months. In comparison, the situation was reversed in steady-state mature communities, where *Erythrobacter* sp. decreased to some 1.6 × 10^8^ 16s rRNA gene copies per m^−2^ in steady-state climax communities, but Chloroflexales increased to populations close to those of cyanobacteria (Figure 3c). The apparent differences in trends between bacteriochlorophyll and 16S rRNA genes as proxies for population size can be explained by the relatively low bacteriochlorophyll content of *Erythrobacter* spp. compared to members of the Chloroflexales [51,52], which essentially made the total content of bacteriochlorophyll be very sensitive to the population size of the latter. 

### 3.3. Cyanobacterial Succession: Diversity and Composition

Unexpectedly, cyanobacterial richness gauged by the number of observed amplicon sequence variants (ASVs) was not significantly different across time points and when compared to steady-state climax communities (Kruskal–Wallis, *p* = 0.33; Table 1), whereas ASV evenness (measured as Pielou’s Evenness) decreased significantly (Kruskal–Wallis, *p =* 0.04) with time. Pairwise Kruskal–Wallis comparisons indicated that the difference was driven by a drop in evenness between early (3 and 6 months) and late succession communities (9 month and steady-state climax) (adjusted *p* = 0.07 for all four comparisons). Shannon’s diversity also followed the evenness trend, with significant differences with time (Kruskal–Wallis, *p* = 0.02) where late succession samples were less diverse than early succession samples (adjusted *p* = 0.06 for all four comparisons). Regarding cyanobacterial community composition (beta-diversity), all time points and steady-state climax communities were significantly different from each other (PERMANOVA, *p* < 0.05, pairwise Kruskal–Wallis *p* < 0.05), a result also supported statistically by a PCOA (principal coordinates ordination analysis; Weighted UniFrac metric; Figure S2).

### 3.4. Identification of Endolithic Cyanobacteria Clades 

In nonporous virgin substrates, only euendolithic organisms can colonize and grow to large abundance. Since we removed all epilithic biomass before sequencing, those organisms found to be abundant early on can be deemed to be bona fide euendoliths since they must have been able to excavate the substrate. Therefore, to identify pioneer euendolithic cyanobacterial clades, the most abundant cyanobacterial ASVs from the 3-month-old tiles were placed using the RAxML Evolutionary Placement Algorithm into the Cydrasil reference cyanobacterial 16S rRNA gene tree containing 980 curated cyanobacterial sequences, which includes all full-length 16S rRNA gene sequences traceable to known euendolithic cyanobacteria (Figure 3d and Figure S3). Euendolithic sequences that were not full length were included in the query sequence list and checked for correlation with known clades. In order to pare down the dataset for placement, we ranked each sample’s cyanobacterial ASVs in order of abundance until cumulative counts reached 95% of the total abundance in each sample, yielding 213 unique ASVs across all tile samples and steady-state climax communities. We then placed the resulting pared ASV dataset into the Cydrasil reference tree. Of the 213 initial ASVs, 139 were placed with high confidence and clustered onto four distinct tree nodes. Two of the nodes contained known euendolithic species: Cluster 2 (containing 37 unique ASVs) encompassed endolithic members in the Pleurocapsales, and Cluster 3 (27 ASVs) contained *Mastigocoleus testarum*. The other two did not align with known euendoliths: one contained *Leptolyngbya* species (Cluster 1; 60 ASVs) and the other was a novel clade that contained only environmental sequences lacking taxonomic assignment and only distantly related (<95.2% similarity) to *Stanieria cyanosphaera*. We named this clade UBC (15 ASVs), for “unknown boring cluster”. 

### 3.5. Colonization Dynamics of Euendolithic Cyanobacterial Clades

To quantify colonization dynamics, qPCR-normalized abundances of the euendolithic clusters were plotted over time (Figure 3d and Figure S3). Members of the UBC were double to an order of magnitude more abundant than the other groups after 3 months of exposure, with an average biomass of 9.1 × 10^9^ 16S rRNA gene copies per m^-2^. UBC abundance remained stable throughout the experiment and was not significantly different when compared to steady-state climax communities. Cluster 1 (*Leptolyngbya-*like) population size lagged that of UBC, reaching a maximum after 6 months (1.1 × 10^9^ 16s rRNA gene copies per m^−2^). Clusters 2 (Pleurocapsalean) and 3 (*Mastigocoleus-*like) colonized substrate at the slowest rate, reaching maximum populations after 9 months (8.7 × 10^10^ and 9.9 × 10^9^ 16S rRNA gene copies per m^−2^, respectively). Clusters 2 (Pleurocapsalean) and 3 (*Mastigocoleus-*like) also decreased in abundance in steady-state climax communities.

### 3.6. Differential Abundance Analysis

In order to identify which cyanobacterial colonizers were driving compositional differences between early (3-month) and late (9-month) tiles, we conducted a differential abundance analysis. The most abundant and significant ASVs at both time points were members of the four clades delineated above (Figure S4). At 3 months, representatives of the UBC were three of the four most abundant cyanobacteria (*p* < 0.05), both in total sequence count and in differential relative abundance (fold change) with respect to 9-month communities. The fourth ASV was a member of Cluster 1, allied to *Leptolyngbya*. At 9 months, Cluster 2 (Pleurocapsalean) and Cluster 3 (*Mastigocoleus*-like) sequences were found to be the most differentially abundant with respect to 3-month communities. 

### 3.7. New Pioneer Euendolith Clade

**Figure 4:**
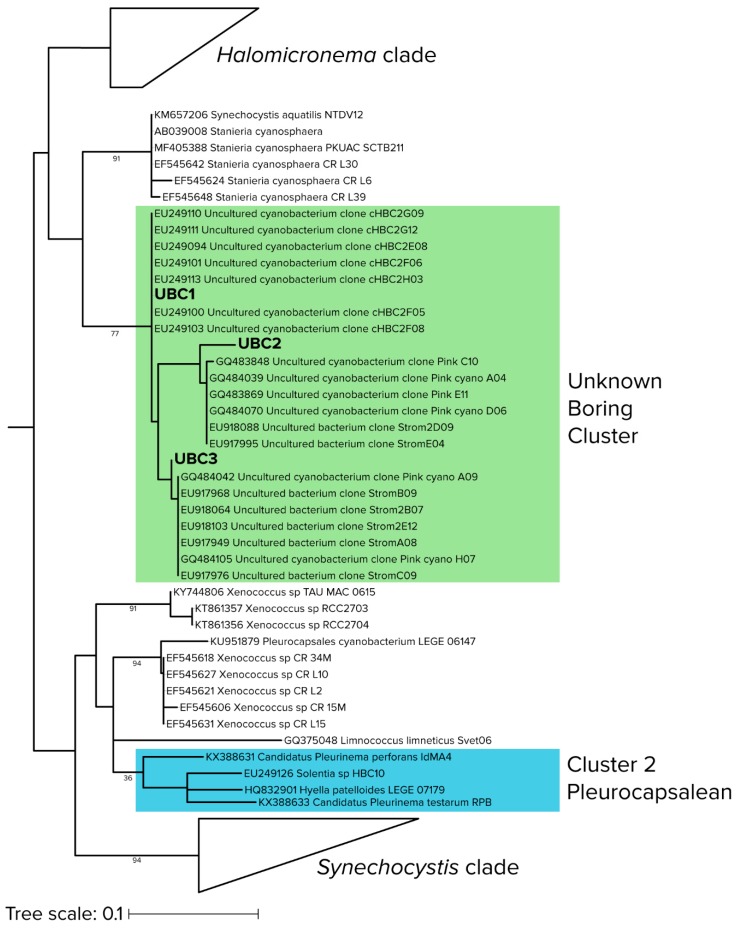
Detailed phylogenetic relationships of sequences in the “unknown boring cluster (UBC)”, with environmental (uncultured) cyanobacterial sequences from stromatolites (shaded in green) and the closest known euendolith cluster (shaded in blue). Branch lengths are substitutions per site and node labels indicate bootstrap values.

Both qPCR-adjusted relative abundance and differential abundance analysis revealed that the previously unknown UBC clade played a significant role in early colonization of hard intertidal carbonates. In order to better constrain its identification, we conducted a maximum-likelihood phylogenetic reconstruction of 395 sequences, largely from cyanobacterial isolates (Figure 4), but including those of the most differentially abundant UBC and the seven sequences most similar to UBC that we could find by BLAST analyses. As before (i.e., Figure 3d), UBC members were only distantly related (<5.2% similar) to cultured cyanobacteria, the nearest being *Stanieria cyanosphaera* (formerly *Chroococcidiopsis cyanosphaera*), an epilithic freshwater unicellular cyanobacterium [53]. UBC was distant from the canonical euendolithic groups, with the Cluster 2 (Pleurocapsalean) being the closest. However, UBC members were phylogenetically close to environmental sequences obtained from marine carbonate microbialites, a habitat not dissimilar from the interior of hard carbonates and containing known euendoliths [54]. 

## 4. Discussion

We recently reported that APBs can be major components of endolithic intertidal ecosystems and could potentially be euendolithic in nature [15], for which no precedent existed. Alternatively, these APBs may constitute secondary colonizers of opened pore space that rely on metabolic interactions with cyanobacteria, as they commonly do in other benthic environments like microbial mats or microbialites [50,54,55]. We hypothesized that examining colonization using molecular techniques and photopigment analysis specifically targeting APBs could help solve this question, in that early colonizers of bare substrates can be logically assumed to be active borers, while a dependency on cyanobacteria should result on delayed colonization by APBs. The temporal dynamics of endolithic population of Chloroflexales indeed suggest that this group of APBs are not euendoliths but instead act as secondary colonizers whose populations do not attain significance until communities of cyanobacteria are mature and the substrate has significantly eroded. The case of the proteobacterium *Erythrobacter* sp. was clearly different, since significant populations of *Erythrobacter* were present early in the colonization process and were sustained through the experimental period. *Erythrobacter* are aerobic anoxygenic phototrophs that conduct photoheterotrophy, have a low BChl *a* content, and require a source of organic carbon [24,56]. Our endolithic sequences were most similar to those in Group I *Erythrobacter* genomes [57]. Under our hypothesis, these organisms could still be euendoliths, even though their populations remained low throughout the experiment. Alternatively, since these small unicellular bacteria are abundant in coastal marine waters [24,56], they could have easily washed into fresh pits made by cyanobacteria in exposed tiles. Our current data cannot fully solve these alternatives. In fact, the metabolic action of photoheterotrophs can increase pH levels around cells, leading the precipitation, not dissolution, of calcium carbonate [15], which would make a boring activity more difficult [58]. By contrast, the lack of the more complex photosynthetic Chloroflexales and low total bacteriochlorophylls suggests that, during colonization, euendolithic cyanobacteria dominate the photosynthetic niche due to their ability to excavate habitable space and utilize the mineral carbon for autotrophy [18,59]. Only once sufficient habitable space has been created by cyanobacteria can significant populations of APBs develop.

We found that the patterns of endolithic cyanobacterial succession within hard intertidal carbonates sustain three distinct phases (early, late succession, and steady-state climax). In our habitat, early colonization is predominantly conducted by a previously undescribed group of euendolithic cyanobacteria (UBC) that rapidly colonizes rock to maximal levels within 3 months. This clade could exceed 40% of endolithic cyanobacterial populations early on. Cluster 1 (*Leptolyngbya-*like) organisms also contribute to early colonization but only reach 60% of the biomass of UBC. By 9 months of incubation, the three canonical groups of euendolithic cyanobacteria, *Leptolyngbya (*which we tentatively equate to the *Plectonema terebrans* morphotype), boring members of the Pleurocapsales, and *Mastigocoleus testarum* gain a foothold. Finally, as the community reaches a steady-state climax composition, euendolithic cyanobacteria are displaced in relative importance by other cyanobacteria and by significant populations of Chloroflexalean APBs. The initial large abundance of the UBC could be explained by the presence of fast-growing propagules in natural seawater that quickly attach and bore into the substrate. Since boring microorganisms are fixed in place in their boreholes, competition for space, which can influence patterns of distribution in benthic cyanobacterial communities [60] is likely not a relevant factor until significant proportions of the rock surface become colonized. Hence, having an early foothold on the substrate may have ensured their persistence through time, as we observed. However, UBC did not continue to increase in population size through the colonization, unlike the total cyanobacterial population, which did. The dynamics of the Cluster 1 (*Leptolyngbya*-like) members were not very different from those of UBC, although they seemed to sustain net population losses in late stages of colonization. The net gains in later stages can be attributed to Cluster 3 (*Mastigocoleus*) and, even more so, Cluster 2 (Pleurocapsalean) cyanobacteria (Figure 3d). As these slow colonizers begin to excavate more carbonate, they could reach a threshold where individual pore spaces become connected and pioneer organisms are no longer fully insulated from competition for space. Chlorophyll and qPCR data suggest that this carrying capacity is reached by 9 months of incubation. This density-dependent competition would also explain the overall decline in cyanobacterial evenness/Shannon diversity with successional progress. Finally, at maturity, as endolithic space has been colonized and the rock has become porous, nonboring endoliths can begin to colonize. One can imagine a scenario where nonboring endoliths, which need not spend energy for excavation, can outcompete borers in the outermost sections of the rock. Euendoliths would still have a competitive advantage deeper within the rock. This would be consistent with the relative decline of boring cyanobacterial ASVs in steady-state climax communities, as they are better adapted to diffusion-limited conditions. Interestingly, we did not see a difference in cyanobacterial pigment concentrations between the 9-month samples and steady-state climax communities, which suggests that nonboring phototrophs may colonize the upper interior of the rock, shading the deeper euendoliths and contributing to their decline.

A comparison of our results with prior colonization studies shows that there exist similarities, as well as marked differences, with the dynamics of porous, biogenic coral skeletons. For example, early work [4,61] demonstrated the divergence in euendolith composition between shells and inorganic calcites. However, careful consideration must be taken as both substrate composition [9,10,12,14] and water depth [9,10] influence community structure, and, as discussed above, there are substantial differences in methodology. Even bearing those caveats in mind, the fact that all four major euendolithic clades are present after 3 months of colonization corroborates the prior conclusions that cyanobacterial colonization happens swiftly, in as little as 4 weeks, with *Plectonema, Mastigocoleus, Solentia,* and *Hyella* species all present [3,8,9,10]. Interestingly, there are no reports of any *Chroococcidiopsis*-like organism that could potentially represent our UBC. We also found that though *Mastigocoleus* does colonize quickly, it does not reach large abundances until the community approaches a steady-state climax composition, in contrast to the findings from corals where it is one of the first and most abundant pioneer organisms. Our observations on Cluster 1 *Leptolyngbya*-like euendoliths agree with the patterns of *P. terebrans* described by Grange et al. [11]. We find that this cluster peaks in abundance after 6 months, which was also found for coral systems. However, when comparing 9-month Cluster 1 *Leptolyngbya-*like populations to those of steady-state climax communities, we found that Cluster 1 *Leptolyngbya-*like populations were less than 10% of the 9-month totals, whereas in corals, *P. terebrans* remains very abundant through maturity [6]. Cluster 2 Pleurocapsalean euendoliths were not very abundant (sometimes < 1%) in previous colonization experiments and surveys, which was attributed to their alleged susceptibility to grazing by fish and chitons due to their shallow mode of boring [11]. This was clearly not the case in our system, with Cluster 2 Pleurocapsalean organisms being the most abundant euendoliths after 9 months. Perhaps grazing pressure was unusually low in our setting, even though we did see abundant, actively grazing chitons during sampling. Though the abundance of eukaryotic euendoliths are widely reported in coral systems [6,11], we did not find a significant contribution of plastid 16S rRNA genes in our samples, and those that were there were not phylogenetically related to known euendoliths. 

In summary, by applying molecular approaches to euendolithic systems we were able to confirm that Chloroflexalean APBs act as secondary colonizers of marine carbonates, illustrate the complex dynamics of cyanobacterial colonization, and define a new clade of likely euendolithic cyanobacteria, highlighting the differences and similarities in succession dynamics between mineral and biogenic carbonates. Our work provides a first look at the complex colonization dynamics that drive bioerosion on these substrates. 

## Figures and Tables

**Table 1:** Alpha diversity metrics of cyanobacterial endolithic communities in tiles placed in the intertidal zone of Isla de Mona and metrics from geographically similar natural substrate communities on Isla de Mona described by Roush et al. [15].

Timepoint	*n*	Observed ASVs	Pielou’s Evenness	Shannon’s Diversity
3 months	3	78 ± 4	0.74 ± 0.06 ^a^	4.55 ± 0.45 ^a^
6 months	3	98 ± 6	0.79 ± 0.01 ^a^	5.22 ± 0.07 ^b^
9 months	3	73 ± 3	0.60 ± 0.06 ^b^	3.67 ± 0.50 ^c^
Climax	3	69 ± 3	0.62 ± 0.02 ^b^	3.67 ± 0.45 ^c^

Community composition of steady-state climax communities was taken from calcite samples published in Roush 2018. Lower-case letters denote samples not significantly different (α = 0.1). ASVs, amplicon sequence variants.

## Supplementary Materials

The following are available online at https://www.mdpi.com/2076-2607/8/2/214/s1, Figure S1: Rarefaction curves of tile communities, Figure S2: PCoA analysis of bacterial and cyanobacterial community composition, Figure S3: Phylogenetic placement of euendolith clusters and colonization dynamics, Figure S4: ASV differential abundance analysis of 3-month and 9-month colonized tiles. Table S1: BIOM tables containing sequences counts for each euendolith cluster at all 3 time points and steady-state climax communities.
