# Supplementary material for: Succession and Colonization Dynamics of Endolithic Phototrophs within Intertidal Carbonates
Source: Microorganisms. 2020 Feb 5;8(2):214. doi: 10.3390/microorganisms8020214 (PMC7074784; doi:10.3390/microorganisms8020214)
Supplement: Supplementary file 1 [file microorganisms-08-00214-s001.zip › supplemental/roush-tiles-supplemental-figures-1-30-2020-resub.docx]

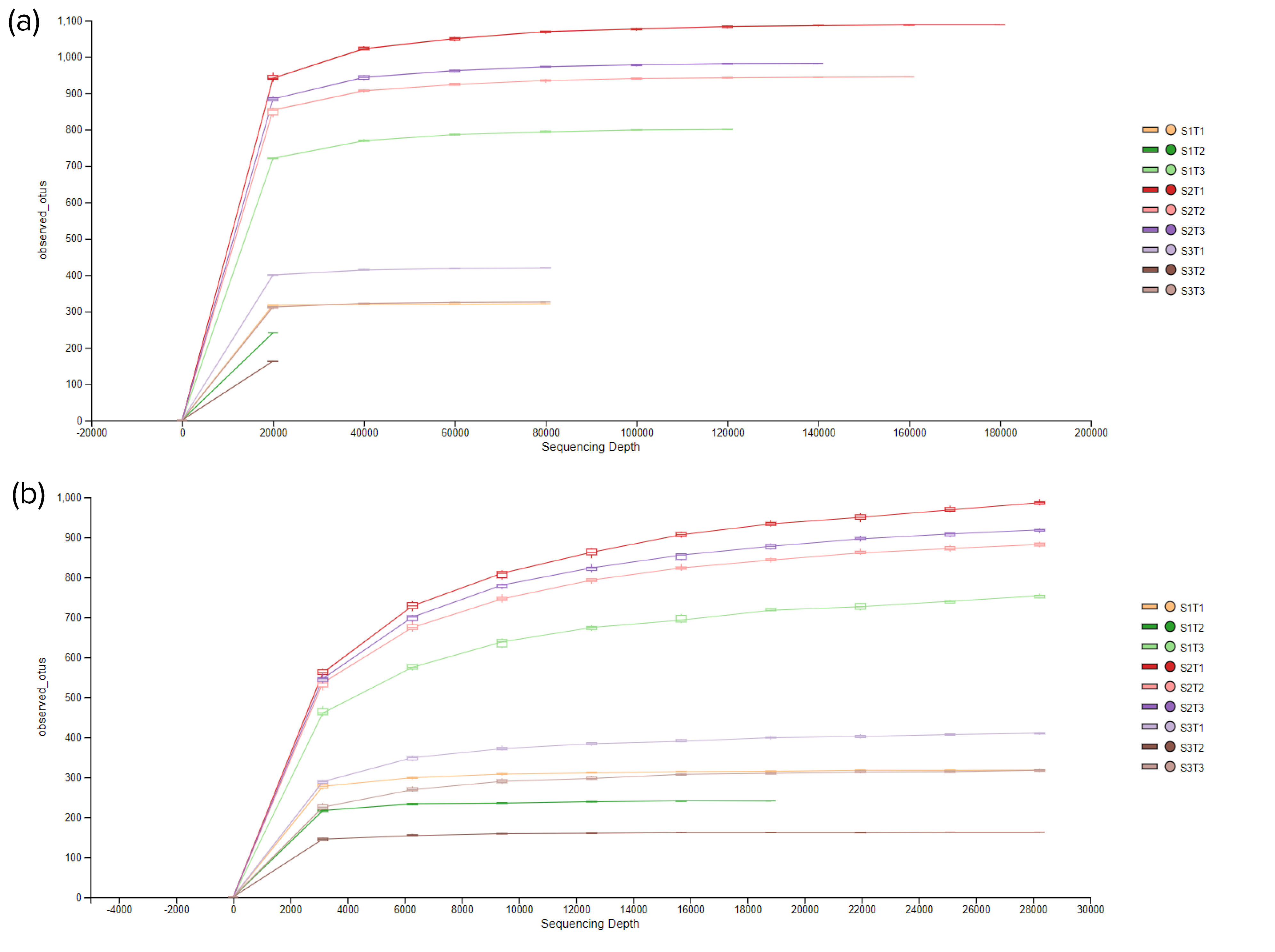


**Figure S1.** Alpha rarefaction curves of observed otus (Unique ASVs) at 180,000 (**a**) and 28,233 (**b**) sequencing depth. All samples reached convergence at their respective maximum sequencing depth.


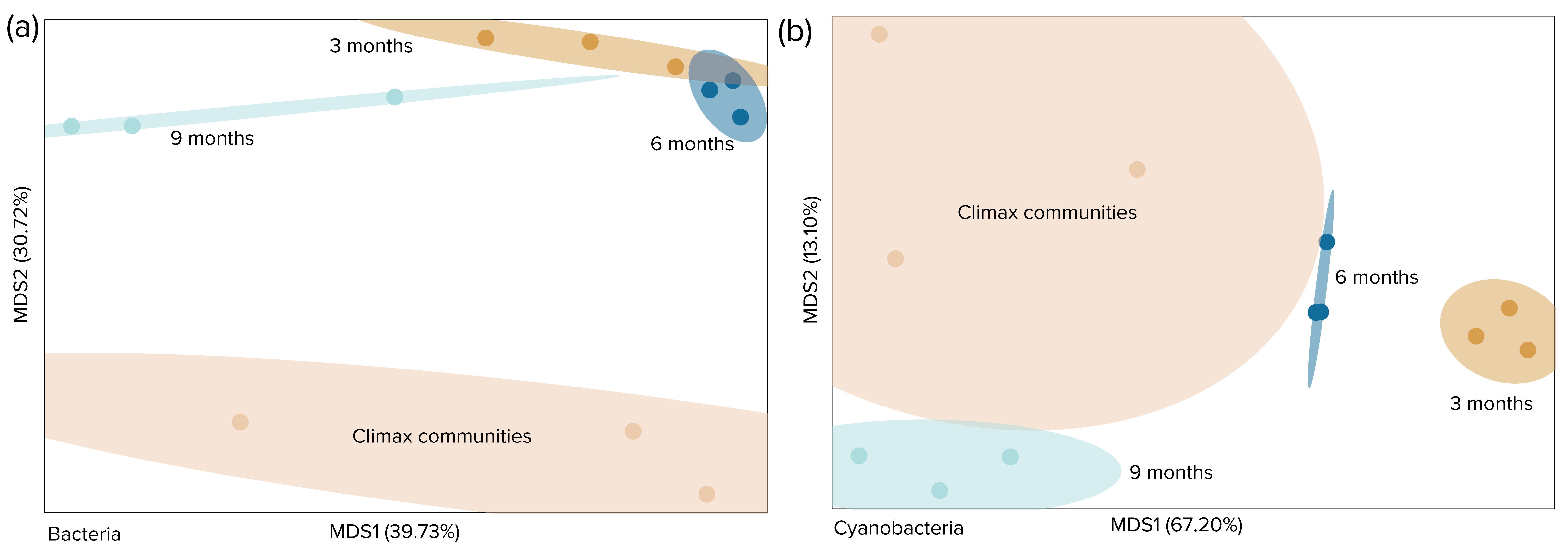


**Figure S2**. Principle coordinates analysis based on the weighted UniFrac metric of all bacteria (**a**) and all cyanobacteria (**b**) both showed that community composition was significantly different between all time points (PERMANOVA, p < 0.05).


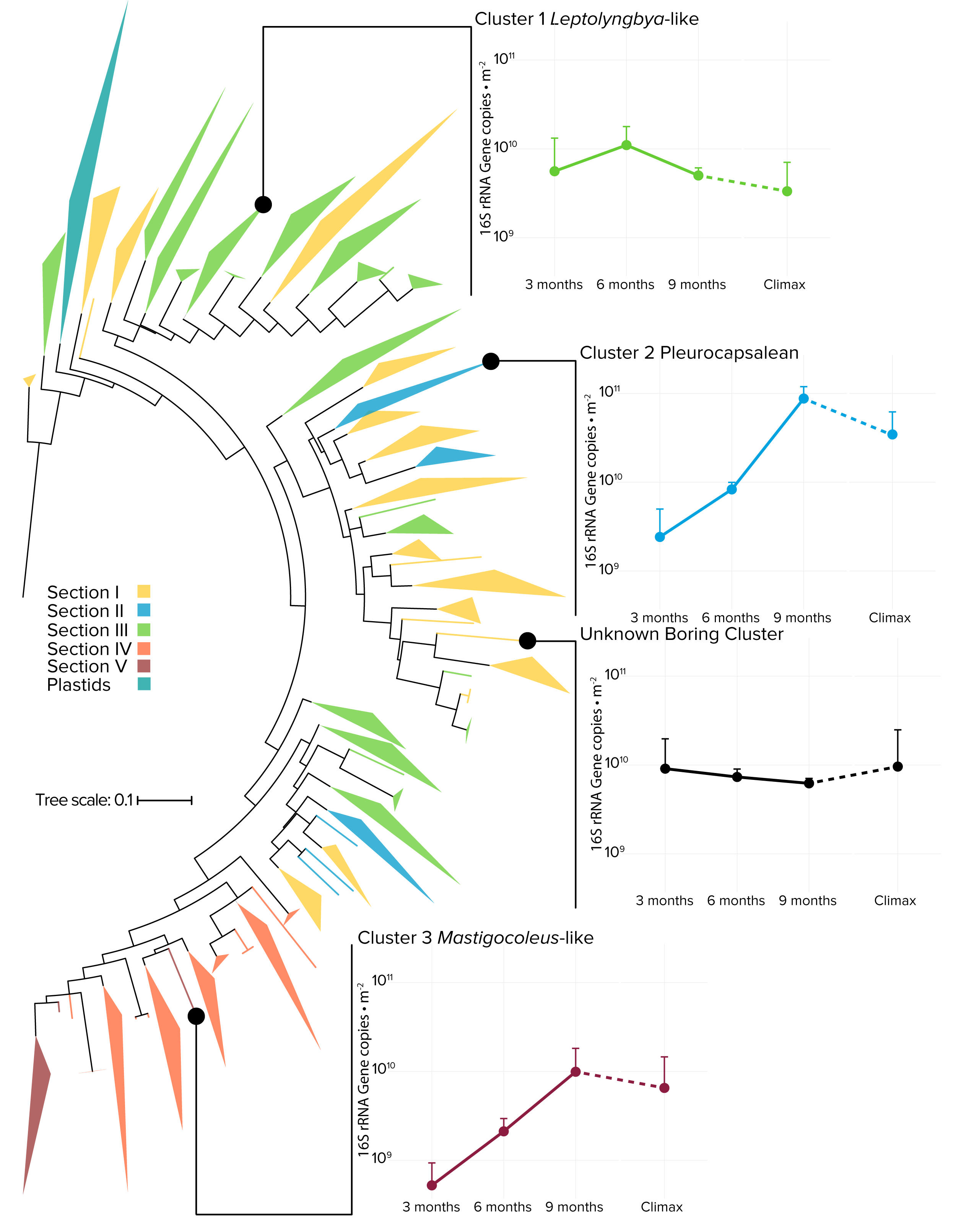


**Figure S3**. Endolithic colonization dynamics of specific microboring cyanobacterial clades, based on qPCR and bioinformatic placement of high-throughput environmental sequences using the Cydrasil cyanobacterial reference tree and database (left, colored by traditional morphotypical sections, *sensu* Rippka 1979 [1]). Error bars are for biological sample triplicates.


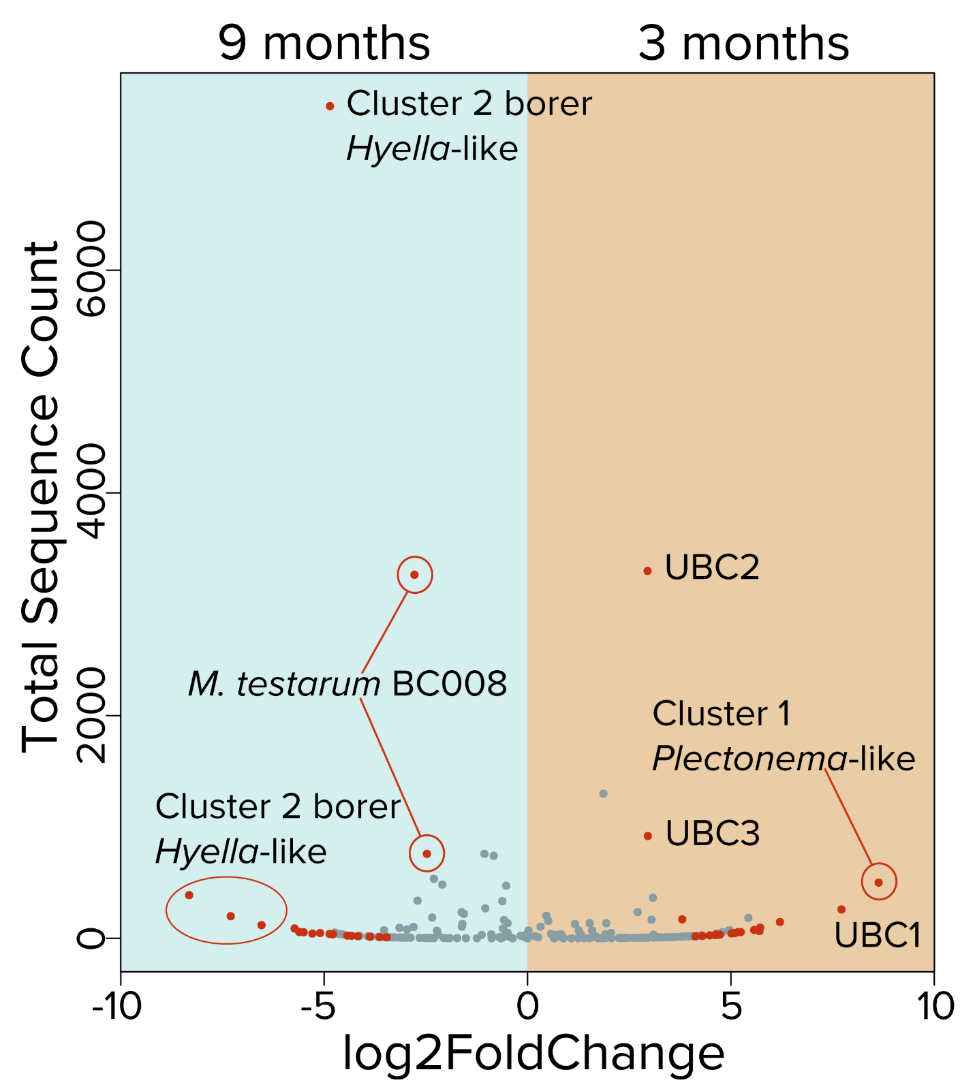


**Figure S4**. Volcano plot of cyanobacterial ASVs comparing differential abundance between tiles incubated for 3 months and 9 months. Red data points are significantly different (*p* < 0.05) between time points. DESeq2 analysis showed the presence of three previously unidentified ASVs not attributable to any known euendolithic genus (UBC).

1. Rippka, R.; Deruelles, J.; Waterbury, J.B. Generic assignments, strain histories and properties of pure cultures of cyanobacteria. *J. Gen. Microbiol.* **1979**, *111*, 1–61, DOI:10.1099/00221287-111-1-1.
